# Supplementary material for: Pioneer Tree Bellucia imperialis (Melastomataceae) from Central Amazon with Seedlings Highly Dependent on Arbuscular Mycorrhizal Fungi
Source: J Fungi (Basel). 2023 May 2;9(5):540. doi: 10.3390/jof9050540 (PMC10219434; doi:10.3390/jof9050540)
Supplement: Supplementary file 1 [file jof-09-00540-s001.zip › Figure S1.pdf]

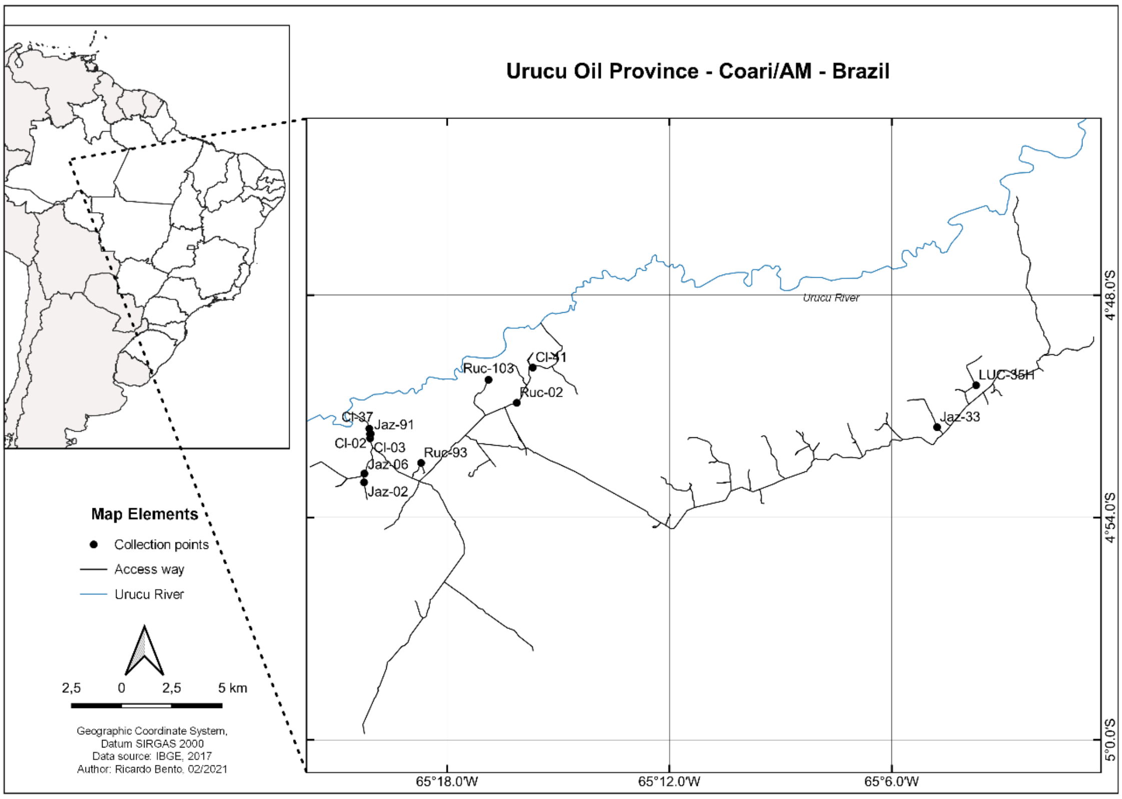

Figure S1. Twelve reforested areas belonging to Petrobras S.A. in the Oil Province of Urucu (PPU), Coari municipality, Amazonas state, Brazil where rhizospheric soil samples used as an inoculant in the treatment with native AMF species were taken. Image-based on Datum WGS 84 to SIRGAS 2000.
