# Supplementary material for: Pioneer Tree Bellucia imperialis (Melastomataceae) from Central Amazon with Seedlings Highly Dependent on Arbuscular Mycorrhizal Fungi
Source: J Fungi (Basel). 2023 May 2;9(5):540. doi: 10.3390/jof9050540 (PMC10219434; doi:10.3390/jof9050540)
Supplement: Supplementary file 1 [file jof-09-00540-s001.zip › Figure S2.pdf]

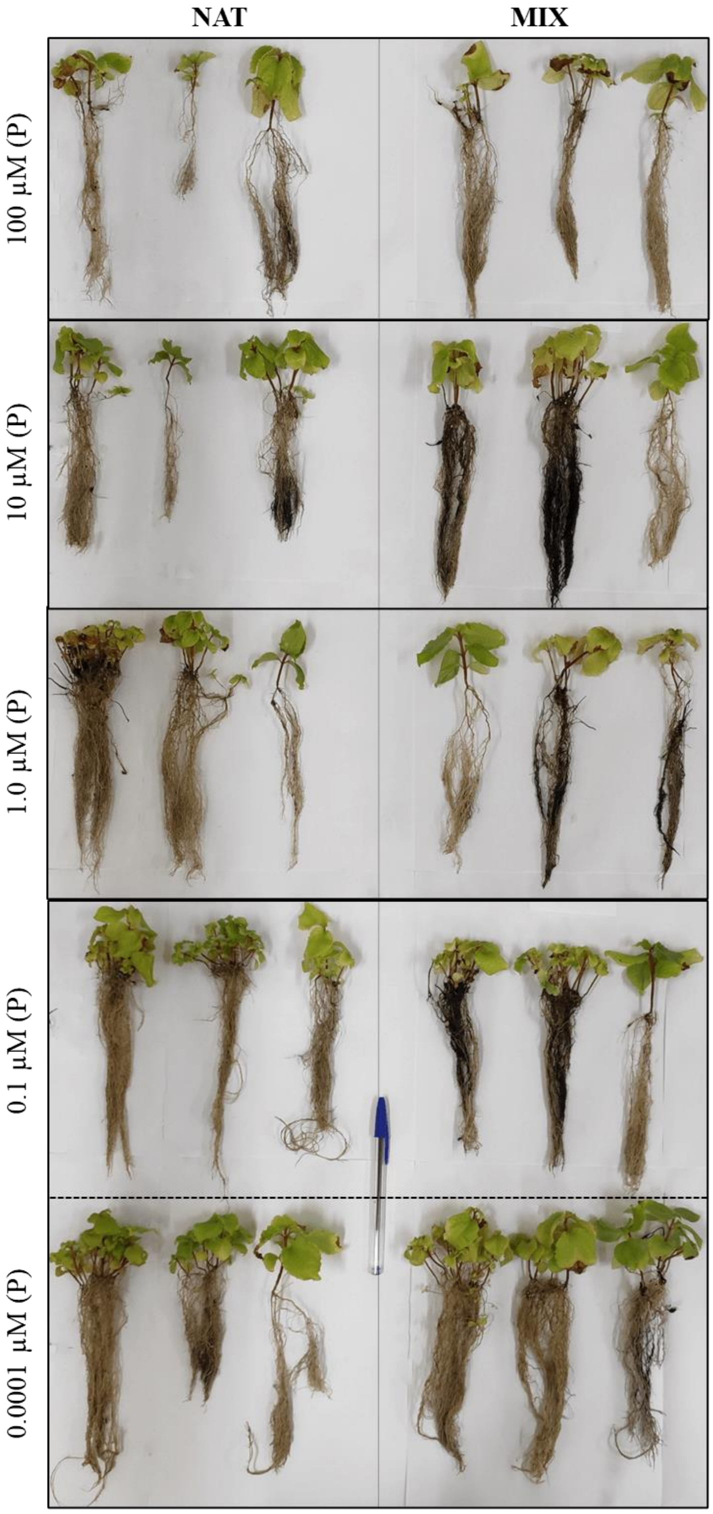

Figure S2. *B. imperialis* seedlings at harvest after 6 months of conducting the experiment in response to different phosphorus dosages (in rows) under two inoculation treatments of arbuscular mycorrhizal fungi (in columns). The NAT column shows the shoot and root biomass of seedlings inoculated with a mixture of native AMF. The MIX column shows the shoot and root biomass of seedlings inoculated with a mixture of AMF from the Fungus Collection of Embrapa Agrobiology. The pen in the picture represents a dimension of 15 cm in length
