# Supplementary material for: Pioneer Tree Bellucia imperialis (Melastomataceae) from Central Amazon with Seedlings Highly Dependent on Arbuscular Mycorrhizal Fungi
Source: J Fungi (Basel). 2023 May 2;9(5):540. doi: 10.3390/jof9050540 (PMC10219434; doi:10.3390/jof9050540)
Supplement: Supplementary file 1 [file jof-09-00540-s001.zip › Table S1.pdf]

Table S1. Chemical composition and the final concentration of modified Hoagland and Arnon (1950) nutrient solution to one-tenth of strength and different P levels for mycorrhizal dependency experiment.

Stock solution	Reagent	Chemical composition	Concentration in the nutrient solution (μM)	Pipetted volume ($mL.L^{-1}$) of Stock solutions at different levels of P				
				0.0001 μM	0.1 μM	1 μM	10 μM	100 μM
1	Potassium phosphate	KH_2PO_4	0–100	0.00001	0.01	0.1	1.0	10.0
2	Potassium chloride	KCl	500–600	6.0	6.0	6.0	5.9	5.0
3	Ammonium sulfate	$(NH_4)_2SO_4$	380	3.8	3.8	3.8	3.8	3.8
4	Calcium nitrate	$Ca(NO_3)_2.4H_2O$	380	3.8	3.8	3.8	3.8	3.8
5	Magnesium sulfate	$MgSO_4.7H_2O$	200	2.0	2.0	2.0	2.0	2.0
6	EDTA-Iron	$C_{10}H_{12}FeN_2NaO_8$	6.13	0.3	0.3	0.3	0.3	0.3
7	Boric acid	H_3BO_3	4.63	0.1	0.1	0.1	0.1	0.1
	Manganese chloride	$MnCl_2.4H_2O$	0.915					
	Zinc sulfate	$ZnSO_4.7H_2O$	0.077					
	Copper sulfate	$CuSO_4.5H_2O$	0.032					
	Cobalte chloride	$CoCl_2.6H_2O$	0.010					
	Sodium molybdate	$Na_2MoO_4.2H_2O$	0.050					
8	MES buffer*	$C_6H_{13}NO_4S$	500	1.0	1.0	1.0	1.0	1.0

*4-morpholine ethanesulfonic acid. The pH of the solution was adjusted to 6.5
