# Supplementary material for: Pioneer Tree Bellucia imperialis (Melastomataceae) from Central Amazon with Seedlings Highly Dependent on Arbuscular Mycorrhizal Fungi
Source: J Fungi (Basel). 2023 May 2;9(5):540. doi: 10.3390/jof9050540 (PMC10219434; doi:10.3390/jof9050540)
Supplement: Supplementary file 1 [file jof-09-00540-s001.zip › Table S2.pdf]

Table S2. Georeference[1] of twelve reforested areas belonging to Petrobras S.A. in the Oil Province of Urucu (PPU), Coari municipality, Amazonas state, Brazil where rhizospheric soil sample used as an inoculant in the treatment with native AMF species were taken.

Areas[2]	Latitude (S)	Longitude (W)	Altitude
CL-02	4°51'44.9"	65°20'04.8"	64 m
CL-03	4°51'51.8"	65°20'04.7"	79 m
CL-37	4°51'36.1"	65°20'06.0"	75 m
CL-41	4°49'57.3"	65°15'41.3"	67 m
JAZ-02	4°53'03.1"	65°20'14.5"	71 m
JAZ-06	4°52'48.9"	65°20'13.9"	70 m
JAZ-33	4°51'33.8"	65°04'46.6"	57 m
JAZ-91	4°51'44.5"	65°20'03.8"	68 m
LUC-35H	4°50'26.0"	65°03'43.5"	71 m
RUC-02	4°50'54.3"	65°16'07.2"	74 m
RUC-103	4°50'17.0"	65°16'52.9"	61 m
RUC-93	4°52'31.7"	65°18'42.1"	78 m

[1]: Georeferences collected with GPS Garmin 70 CSx, Datum WGS 84.

[2]: The areas were divided into three categories: (1) Drilling areas (LUC-35H, RUC-02, RUC-93, and RUC-103) where oil exploration and prospecting are carried out with reforested areas around; (2) Deposit areas (JAZ-02, JAZ-06, JAZ-33, and JAZ-91) opened with the purpose of serving as a deposit for leftover construction materials as well as borrow areas; and (3) Areas of clearings (CL-02, CL-03, CL-37, and CL-41) are those strategically opened along the access branch (roads) of the oil prospecting areas to serve as a deposit of materials for civil construction and the necessary engineering for drilling oil wells.
