# Supplementary material for: Pioneer Tree Bellucia imperialis (Melastomataceae) from Central Amazon with Seedlings Highly Dependent on Arbuscular Mycorrhizal Fungi
Source: J Fungi (Basel). 2023 May 2;9(5):540. doi: 10.3390/jof9050540 (PMC10219434; doi:10.3390/jof9050540)
Supplement: Supplementary file 1 [file jof-09-00540-s001.zip › Table S3.pdf]

Table S3. ANOVA results for the variables Shoot Fresh Mass (SFM), Leaf Area (LA), Shoot Dry Mass (SDM), Root Fresh Mass (RFM), Mycorrhizal Colonization (MC); Number of Mycorrhizal Spores (NMS), and Shoot P Concentration (SPC). Predictor variables are the different phosphorus dosages (Doses), AMF inoculation treatments (Inoc), and their interaction (Doses:Inoc).

Response	Predictor	Df	Sum Sq	Mean Sq	F value	Pr(>F)	CV (%)
SFM	Doses	4	4.58	1.145	5.778	0.00294	40.53
	Inoc	1	0.044	0.0441	0.222	0.64227	
	Doses: Inoc	4	0.702	0.1754	0.885	0.49069	
	Residuals	20	3.963	0.1982			
LA	Doses	4	4672	1168.1	11.169	0.00010	34.78
	Inoc	1	112	112.3	1.074	0.31200	
	Doses: Inoc	4	550	137.4	1.314	0.29900	
	Residuals	20	2092	104.6			
SDM	Doses	4	0.25025	0.06256	5.853	0.00275	44.76
	Inoc	1	0.0032	0.0032	0.3	0.59015	
	Doses: Inoc	4	0.03261	0.00815	0.763	0.56180	
	Residuals	20	0.2138	0.01069			
RFM	Doses	4	9.5	2.375	3.153	0.03660	46,34
	Inoc	1	2.46	2.4596	3.265	0.08580	
	Doses: Inoc	4	3.527	0.8818	1.171	0.35350	
	Residuals	20	15.066	0.7533			
MC	Doses	4	2180	545	2.439	0.08053	31.41
	Inoc	1	3529	3529	15.788	0.00075	
	Doses: Inoc	4	1705	426	1.908	0.14854	
	Residuals	20	4470	223			
NMS	Doses	4	391405	97851	0.584	0.67800	111.77
	Inoc	1	1101317	1101317	6.57	0.01850	
	Doses: Inoc	4	635456	158864	0.948	0.45720	
	Residuals	20	3352753	167638			
SPC	Doses	4	4.332	1.0829	6.542	0.00155	39,37
	Inoc	1	0.847	0.8467	5.115	0.03502	
	Doses: Inoc	4	0.916	0.229	1.383	0.27539	
	Residuals	20	3.311	0.1655			

Degrees of freedom (Df), Sum of squares (Sum Sq), Mean square (Mean Sq) and P-value (Pr>F) of F test. CV (%) - Coefficient of Variation.
